# Hyaluronic Acid Facilitates Angiogenesis of Endothelial Colony Forming Cell Combining With Mesenchymal Stem Cell *via* CD44/ MicroRNA-139-5p Pathway

**DOI:** 10.3389/fbioe.2022.794037

**Published:** 2022-03-08

**Authors:** Yufang Luo, Fang Liang, Xinxing Wan, Shengping Liu, Lanfang Fu, Jiake Mo, Xubiao Meng, Zhaohui Mo

**Affiliations:** ^1^ Department of Endocrinology, Third Xiangya Hospital of Central South University and Diabetic Foot Research Center of Central South University, Changsha, China; ^2^ Department of Endocrinology, Haikou People’s Hospital and Haikou Affiliated Hospital of Central South University Xiangya School of Medicine, Haikou, China; ^3^ School of Medicine, Hunan Normal University, Changsha, China

**Keywords:** hyaluronic acid, endothelial colony-forming cells, mesenchymal stem cells, angiogenesis, CD44, miR-139-5p

## Abstract

Stem cells and progenitor cells have been identified as potential new therapeutic options for severe limb ischemia to induce angiogenesis, and hyaluronic acid (HA) is commonly applied as a biomaterial in tissue engineering. However, the efficiency of HA combined with human umbilical cord blood-derived endothelial colony forming cells (ECFCs) and human umbilical-derived mesenchymal stem cells (MSCs) on angiogenesis is unclear. In the present study, we showed that HA promoted angiogenesis induced by MSCs-ECFCs in Matrigel plugs and promoted blood perfusion of murine ischemic muscles. Laser confocal microscopy revealed that human-derived cells grew into the host vasculature and formed connections, as shown by mouse-specific CD31^+^/human-specific CD31^+^ double staining. *In vitro* assays revealed that HA supported cell proliferation and migration, enhanced CD44 expression and reduced microRNA (miR)-139-5p expression. Further analysis revealed that miR-139-5p expression was negatively regulated by CD44 in ECFCs. Flow cytometry assays showed that HA increased CD31 positive cells proportion in MSC-ECFC and could be reversed by miR-139-5p mimics transfection. Moreover, the improvement of MSC-ECFC proliferation and migration induced by HA could be blocked by upregulation of miR-139-5p expression. In conclusion, HA facilitates angiogenesis of MSCs-ECFCs, and this positive effect be associated with activation of the CD44/miR-139-5p pathway, providing a promising strategy for improving severe limb ischemia.

## Introduction

Peripheral arterial disease (PAD) is a progressive disorder characterized by stenosis and/or occlusion of large and medium-sized arteries, most of which occur in the lower extremities. It is estimated that more than 200 million patients suffer from PAD worldwide (Shu and Santulli, 2018). Patients with PAD suffer an increased risk of coronary and cerebrovascular mortality and morbidity (Criqui and Aboyans, 2015), especially those with diabetes (Fadini et al., 2020). PAD progresses to a more severe disease stage without appropriate treatment: critical limb ischemia (CLI). The 5-year survival rate of CLI is less than 50% (Gao et al., 2019). Traditional treatment strategies are aimed at restoring perfusion of the ischemic limb and involve surgical bypass or endovascular interventions. But as 40% of patients miss the chance for surgical revascularization and intervention (Das, 2009), improving therapeutic measures for PAD is necessary.

It has been suggested that quickly restoring blood flow is essential to save organs, and cell therapy may be a promising strategy for PAD (Creager et al., 2012; Frangogiannis, 2018). Endothelial colony forming cells (ECFCs), also termed late outgrowth endothelial precursor cells (EPCs), display high clonogenic potential and can originate *de novo* blood vessels *in vivo*. ECFC transplantation has been shown to improve various ischemic disorders, including PAD, and these cells are considered a promising tissue-engineered material (Faris et al., 2020). Further evidence revealed that the regenerative efficacy of ECFCs was enhanced when they were transplanted in combination with mesenchymal stem cells (MSCs), a kind of pluripotent stem cell that can differentiate into perivascular mural cells (Lin et al., 2014; Rossi et al., 2017). In addition to directly participating in vascular construction, MSCs can secrete growth factors, anti-inflammatory factors and cytokines and therefore improve the efficiency and safety of ECFC transplantation (Kang et al., 2017; Souidi et al., 2017). However, the application of cell therapy is challenged by limited engraftment of cells after transplantation; factors such as exposure of the cells to ischemia and inflammation, mechanical washout of cells from the vasculature and leaking of the cell suspension from the targeted injection site contribute to poor cell retention (Robey et al., 2008).

To improve the hostile microenvironment for resident stem cells, we have tried to optimize the administration method of cells. The application of extracellular matrix has attracted attention due to its biodegradable and biocompatible properties. Of note, hyaluronic acid (HA) is one of the most abundant components of the extracellular matrix. Composed of repeating polymeric glucuronic acid and N-acetyl-glucosamine disaccharides, HA is abundantly expressed in several tissues. Currently, HA is commonly applied as a biomaterial in tissue engineering (Bonafè et al., 2014; López-Ruiz et al., 2019). HA-based hydrogel-embedded EPCs promoted angiogenesis in ischemic limbs (Ratliff et al., 2010) and in ischemic myocardium (Gaffey et al., 2015; Gaffey et al., 2019) compared with EPC administration alone. However, the underlying mechanism is unclear. Recently, our team reported that HA combined with ECFCs-MSCs accelerated healing of refractory diabetic foot ulcers (Zhao et al., 2020). However, the efficiency of HA-MSC-ECFC combined therapy on angiogenesis in ischemic disorders is unclear.

As an important ligand of several membrane receptors activating intracellular signaling cascades, HA and its interactions with the transmembrane receptor CD44 play an essential role by modulating cellular growth, development, adhesion, and migration (Gupta et al., 2019). High CD44 expression on ECFCs is a trophic factor in neovasculature (Sakimoto et al., 2017). As one of the major cell surface HA receptors, CD44 has been proven to be tightly correlated with the development of multiple malignances by regulating microRNAs (miRs) (Bourguignon et al., 2010; Bourguignon et al., 2012a; Bourguignon et al., 2012b). In ischemic kidneys, CD44-targeted HA combined with adipose-derived MSCs decreased the expression of miR-21, miR-17-5p and miR-10a, miRs involved in the inhibition of inflammation and apoptosis during renal ischemia (Awadalla et al., 2021). Recently, miR-139-5p was reported to play a vital role in regulating ECFC function (Luo et al., 2020). To date, no evidence exists on the role of CD44/miR-139-5p pathway in tissue regeneration and angiogenesis. We wonder whether HA promotes MSC-ECFC angiogenesis through CD44/miR-139-5p signaling.

Here, we show that HA facilitates angiogenesis of ECFCs combined with MSCs and reveal the underlying mechanism by which the CD44/miR-139-5p pathway participate in this process.

## Materials and Methods

### Cell Isolation and Characterization

The study was approved by the Third Xiangya Hospital Review Board. ECFCs were isolated from human umbilical cord blood samples as described previously (Luo et al., 2020). Briefly, human umbilical cord blood samples (40 ml) were collected in heparinized solution. The mononuclear cell fraction was isolated by density gradient fractionation, resuspended in complete EGM‐2 medium (Lonza, Rockland, ME, United States), and then plated to 6-well tissue culture plates (5 × 10^7^ cells/well) precoated with type 1 rat tail collagen (BD Biosciences, Bedford, MA, United States). The medium was changed daily for a week and then every other day until the first passage. Isolated ECFCs were cultured to passages four to seven for cellular assays.

MSCs were isolated from human umbilical cord as described preciously (Yue et al., 2020). Briefly, collected umbilical cords were rinsed in sterile saline, and digested at 37°C for 4 h in Dulbecco’s modified Eagle’s medium (DMEM; Gibco, United States) containing 0.1% collagenase I (Sigma-Aldrich Co., United States) after cutting into 2–3 mm sections. The resulting cell suspension was filtered, centrifuged and then cultured in complete low-glucose DMEM medium (FBS; Gibco). Nonadherent cells were removed 2 days after seeding. The medium was changed every other day until the first passage. Isolated MSCs were cultured to passages three to six for cellular assays.

### *In Vivo* Matrigel Plug Assays

All animal studies were approved by the Institutional Animal Care Committee of Central South University. Matrigel (500 µL) containing PBS, HA, ECFCs, HA-ECFCs, MSCs, HA-MSCs, ECFCs-MSCs or HA-ECFCs-MSCs was injected subcutaneously into the abdomen of nude mice (*n* = 3). The total cell number were 5 × 10^6^ cells/gel and the ratio of MSC to ECFC was 3:2. The Matrigel was removed 12 days after transplantation and prepared for paraffin sections (Figure 2A). Post-preparation, the blood vessels were stained with human specific CD31 antibody and examined by microscope (Olympus, Lake Success, NY, United States).

### Hindlimb Ischemia Model and Limb Perfusion Ratio Assessment

A severe model of murine hindlimb ischemia was performed as previously described (Luo et al., 2020). After anesthetizing with 1% pentobarbital intraperitoneal injection, the entire right superficial femoral artery and vein (from just below the deep femoral arteries to the popliteal artery and vein) were ligated with 8–0 silk sutures, cut and excised with an electrical coagulator. The overlying skin was closed with 5–0 silk sutures. After surgery, 40 mice were selected and randomly divided into four groups. Mice in group A received intramuscular injection of PBS, group B received intramuscular injection of HA, group C received intramuscular injection of 5 × 10^6^ MSCs-ECFCs (3:2), and mice in group D were injected with mixture of HA and 5 × 10^6^ MSCs-ECFCs (3:2). Ischemic muscles were collected 14 days after transplantation and then prepared for paraffin sections.

A real-time microcirculation imaging analysis was performed using a PeriCam Perfusion Speckle Imager (PSI) based on the laser speckle contrast analysis technology (Perimed Inc., Kings Park, NY, United States). It was used to evaluate the limb perfusion ratio [ischemic limb (right)/normal limb (left)] at days 0, 7, and 14 after ischemia. Ambulatory impairment was scored using the criteria described previously (Zhang et al., 2018). Skin temperature was measured using a digital thermometer.

### Histological Assessment and Detection of Vascular Density

After antigen retrieval and endogenous peroxidase blockade, the dewaxed paraffin section of tissue was incubated overnight with anti-CD31 mouse monoclonal antibody (1:100, Servicebio, Wuhan, China) or anti-CD31 human polyclonal antibody (1:200, Servicebio, Wuhan, China) at 4°C, respectively. The slide was washed and incubated with goat anti-mouse secondary antibody (1:300, Servicebio, Wuhan, China) or goat anti-rabbit secondary antibody (1:400, Servicebio, Wuhan, China) at room temperature for 2 h. The slide was then subjected to blockage with biotin and avidin (ABC) solution, color development with 3,3′-diaminobenzidine (DAB) solution, dehydration, and sealing. Under normal optical microscope, three high-power fields (400X) were randomly selected for each slice to count vascular density, and three high-power fields (200X) were randomly selected for each slice to count CD31-stained positive area (brown) and vessel size. CD31-stained positive area represented of proportion of cells stained by CD31 antibody, and vascular size measured the area of vascular, which surrounded by CD31^+^ cells. CD31^+^ staining was quantified by ImageJ. Double staining of human specific CD31^+^ and mouse specific CD31^+^ cells were detected and photographed under laser confocal microscopy high-power fields (400x). Morphological changes of muscle tissue were observed by hematoxylin staining under normal optical microscope.

### Cell Proliferation

CCK-8 assays were performed for evaluation of cell proliferation. Briefly, cells were seeded in 96-well plates at a density of 5 × 10^3^ cells/well (co-cultured MSCs-ECFCs at ratio of 3:2) and incubated at 37°C for 24 h. Equal volume of 0.5 mg/ml or 1.0 mg/ml HA dilution, or PBS was added in the medium subsequently. Then 10 μL of CCK-8 solution (Sangon Biotech, Shanghai, China) was added to each well of the plate. After incubation for 2 h, the absorbance at 450 nm was measured.

### *In Vitro* Scratch Assays

Cell migration was evaluated with an *in vitro* scratch assay as previously described (Yue et al., 2020). MSCs, ECFCs and MSCs-ECFCs (at ratio of 3:2) were seeded in six-well plates at a density of 2 × 10^5^ cells/well. The cells were cultured at 37°C for approximately 24 h until full confluence, and a straight-line scratch was made with a 10-μL pipette tip. And then fresh serum-free medium was added. The cells were photographed immediately, 6, 12, 18 and 24 h after scratch. The migration of cells to the scratch bed was quantified using ImageJ software.

### Cell Transfection

miR-139-5p mimic (miR10000250), miR-139-5p inhibitor (miR20000250), NC mimic, NC inhibitor and CD44 siRNA were synthesized by RiboBio (Guangzhou, China), and CD44 plasmids were purchased from Vigene (Jinan, China). The final concentration of miR-139-5p mimic, miR-139-5p inhibitor, NC mimic, NC inhibitor and CD44 siRNA in the transfection system was 100 nM and the concentration of CD44 plasmid in the transfection system was 1ug/ml. Cells were transfected with Lipofectamine 3000 (Invitrogen, Carlsbad, CA, United States) when the confluence was approximately 70–80%. Cell lysate for qPCR assays was collected 48 h after transfection, and for Western blot assays 72 h after transfection, respectively.

### Western Blot Assay

Western blot was performed as previously reported (Luo et al., 2020). Primary antibodies include CD44 (Abcam, Cambridge, United States), VEGF (Cell Signaling Technology, Danvers, MA, United States), PDGF-B (Abcam, Cambridge, United States), Tubulin (Proteintech, Wuhan, China) and GAPDH (Proteintech, Wuhan, China). The immunoblotted proteins were visualized using an ECL Western blotting luminal reagent (Advansta, United States) and quantified using a universal Hood II chemiluminescence detection system (Bio-Rad, United States).

### Real-Time Reverse Transcription Polymerase Chain Reaction (qPCR)

The total RNA was isolated with TRIzol reagent (Life technology) and reverse transcription performed using stem-loop RT primer for miRNA with Superior III RT Supermix (Innogene biotech, Beijing, China). PCR was performed with the SYBR Green I Fast qPCR Mix (Tsingke, Beijing, China). The experiments were performed as per the protocols. Real-time PCR was performed using the Roche LightCycler480 II. The expression was quantified relative to the housekeeping gene (glyceraldehyde-3-phosphate dehydrogenase) for mRNA and U6 for miRNA.

### Flow Cytometry

MSCs and ECFCs at passages three to five were harvested and plated into 6-well plates (2 × 105/well, at ratio of MSCs: ECFCs = 3:2) with EGM-2 medium. For the transfection group, miR-139-5p mimics or NC mimics was transfected 24 h after cell seeded and cells were cultured in the subsequent 48 h. HA was added 24 h before the flow cytometry assays. Cells were digested with 0.25% trypsin-EDTA (Gibco, United States) and 1 × 10^6^ cells were re-suspended in 100 μL PBS, followed by incubating with 5 μL fluorescein isothiocyanate (FITC)-conjugated anti-human CD31 antibodies (eBioscience, United States) on ice in the dark for 30 min. Cytometric analysis was performed using a flow cytometer (BD FACSAria II3, United States) and the results were analyzed using FlowJo (Tree Star Inc.; Ashland, OR, United States).

### Statistical Analysis

Data are expressed as mean ± SD. Two groups were compared by the unpaired Student’s *t*-test and multiple groups were analyzed by 1-way analysis of variance. When the condition of homogeneity of variance is satisfied, Tukey test was used for pairwise comparison between groups. When the variance is uneven and the pairwise comparison between all groups is concerned, the Game-Howell test was used (SPSS 23.0, NY, United States). *p* < 0.05 was considered significant.

## Results

### HA Enhanced the Proliferation and Migration of MSCs and ECFCs

**FIGURE 1:**
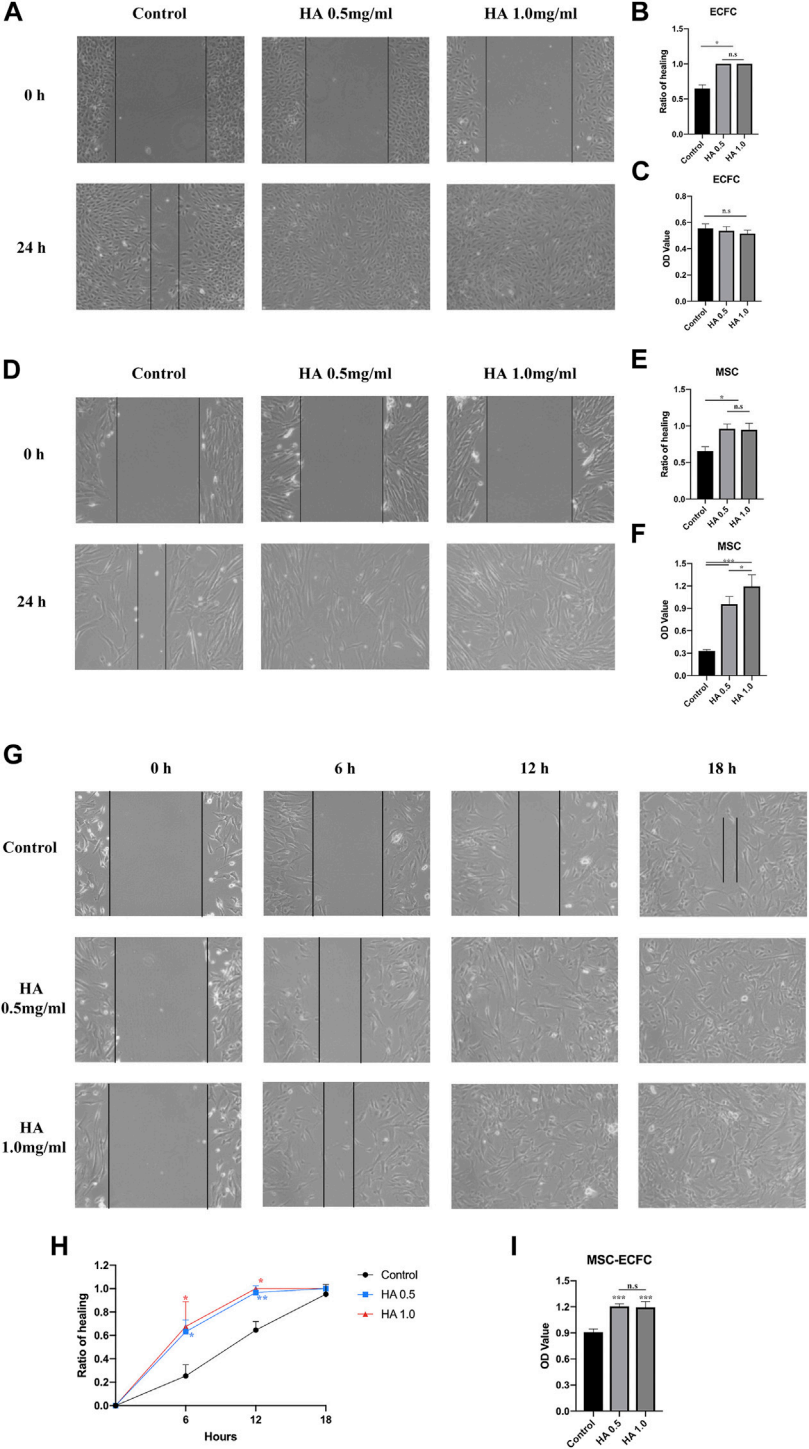
Effect of HA dilutions on cell viability and migration. Following 24 h of exposure to HA, wound healing assays were performed to assess the migration of ECFCs **(A,B)**, MSCs **(D,E)** and MSCs-ECFCs **(G,H)**. CCK-8 assays were performed to assess the proliferation of ECFCs **(C)**, MSCs **(F)**, and MSCs-ECFCs **(I)**. (*n* = 3 independent experiments. Error bars: mean ± SD. *p < 0.05, **p < 0.01, ***p < 0.001).

To investigate the effect of HA on MSCs and ECFCs, we initially analyzed the effect of 0.5 mg/ml or 1.0 mg/ml HA on proliferation and migration. Wound scratch assays showed that MSCs or ECFCs treated with HA dilution displayed significantly accelerated migration and achieved confluence within 24 h, while no differences in migration are observed between two HA concentrations (Figures 1A,B,D,E). CCK8 assays showed that HA promoted the proliferation of MSCs (concentration-dependent), but not that of ECFCs (Figures 1C,F).

Then, we tested the effect of HA dilution on cocultured MSCs-ECFCs. Wound scratch assays showed that HA accelerated the cell migration rate; thus, the HA-treated MSC-ECFC mixed cells achieved confluence within 18 h (Figures 1G,H). These results indicated that MSC-ECFC coculture improved migration compared with MSCs or ECFCs cultured alone, and HA exposure further accelerated the migration rate of MSCs-ECFCs. CCK-8 assays (Figure 1I) showed that HA dilution significantly promoted the proliferation of MSCs-ECFCs. No significant difference in mixed cells migration and proliferation are observed between two HA concentrations. Therefore, we used 0.5 mg/ml concentration for subsequent experiments.

### HA Promoted MSC-ECFC Induced Angiogenesis

**FIGURE 2:**
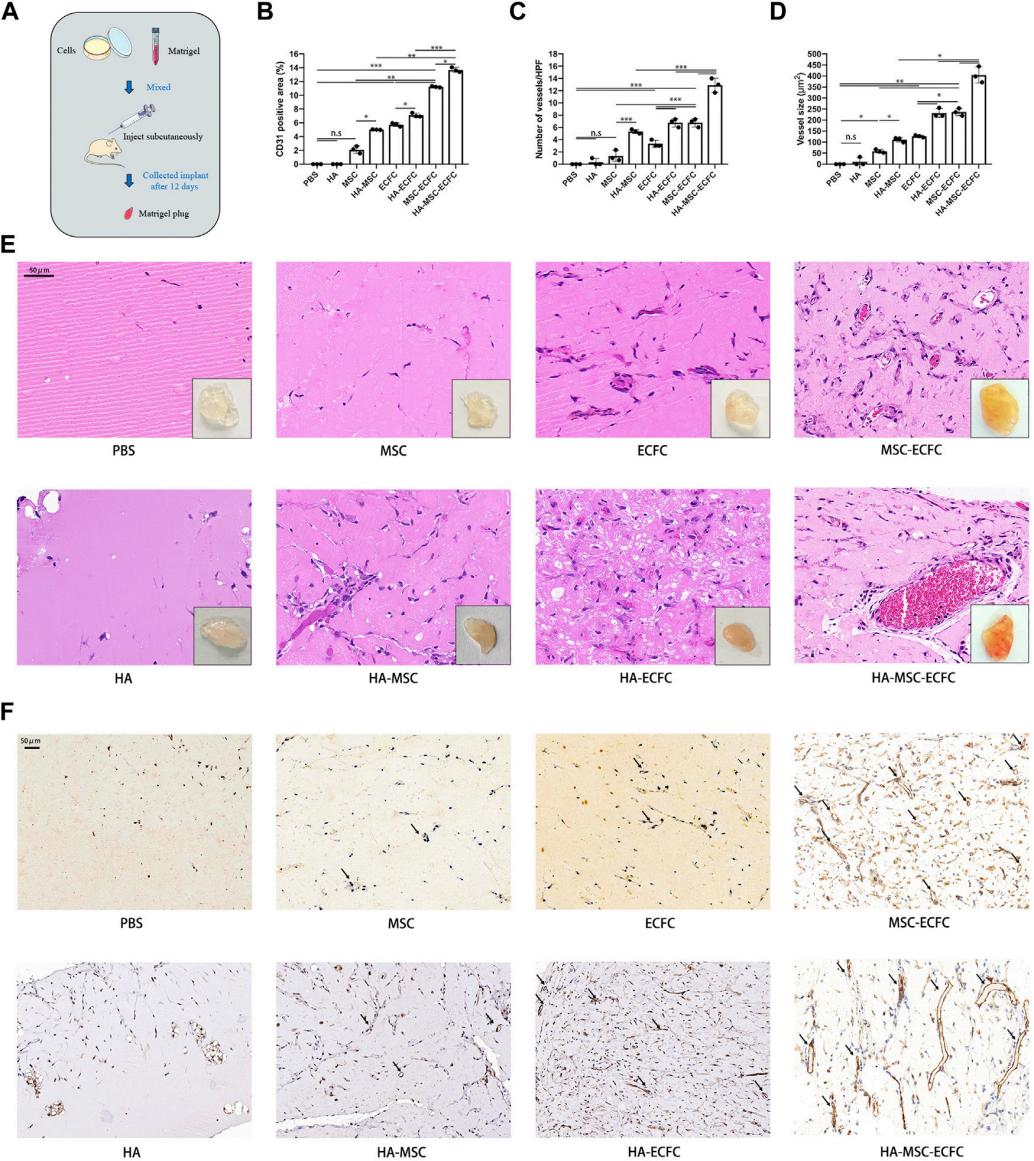
HA promotes MSC-ECFC-induced angiogenesis of Matrigel plugs *in vivo*. **(A)**. The procedure for Matrigel plug *in vivo* assays. **(B–D)**. Quantitation of vessel number from the H&E-stained paraffin sections and quantitation of the human-specific CD31-positive percentage and vessel size. **(E, F)** Representative images of macroscope view of explanted Matrigel plugs, H&E staining (magnification ×400) and immunobiological human CD31 staining (magnification ×200) of explanted Matrigel plugs. (*n* = 3 independent experiments. Scale bar represents 50 μm. Error bars: mean ± SD. *p < 0.05, **p < 0.01, ***p < 0.001).

To test the *in vivo* angiogenesis of HA combined with stem cells, we performed *in vivo* Matrigel plug assays (Figure 2A). Results showed that implants of HA alone were devoid of vessels, indicating that vehicle alone was unable to form vasculature. Implants of MSC alone could generated vascular, however, there were no significant increased compared with control groups. While ECFC plugs contained a certain number of vascular, MSC-ECFC (3:2) cotransplantation significantly increased vessel numbers compared with MSC or ECFC plugs as previous study demonstrated (Melero-Martin et al., 2008). Moreover, we noticed that HA promoted angiogenesis of MSC, ECFC and MSC-ECFC, respectively. Implants of HA-MSC-ECFC were thoroughly reddish in color, indicating abundant blood flow perfusion. H&E staining showed numerous vessels containing erythrocytes in implants containing MSCs-ECFCs. Moreover, vessels with large diameters appeared in the HA-MSC-ECFC plugs. HA cotransplantation with MSCs-ECFCs resulted in additive effects on the vessel density, vessel size and CD31-positive areas (Figures 2B–F). These results demonstrated that *in vivo* angiogenesis induced by MSC-ECFC dual cells is stronger than mono-cell, and HA coadministration further increased the MSC-ECFC-induced vascular network.

### HA Promoted Angiogenesis of MSCs-ECFCs and Blood Flow Restoration of Ischemic Hindlimb in Mice

**FIGURE 3:**
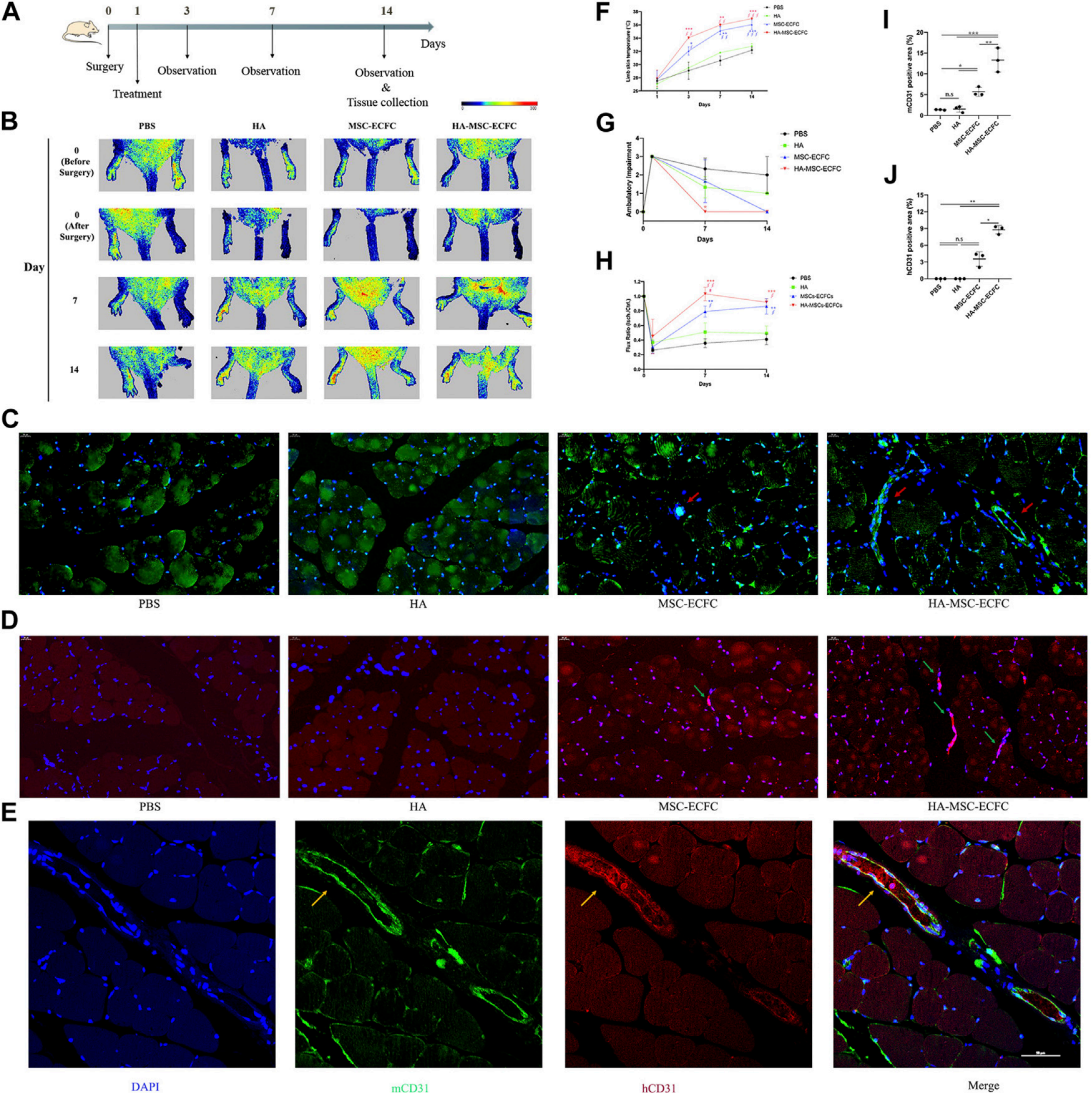
HA improves MSC-ECFC-induced blood flow recovery of HLI in mice. **(A)**. The process of generating mouse models of hindlimb ischemia. **(B)**. Improvements in blood flow recovery were evaluated using PSI analysis in the ischemic limbs of 8-week-old mice injected with PBS, HA, MSCs-ECFCs or HA-MSCs-ECFCs before surgery and 0-, 7- and 14-days post-surgery. **(C,D)**. Representative fluorescent images of mouse-specific CD31^+^ tissue **(C)** and human-specific CD31^+^ tissue **(D)** at 14 days after surgery (magnification ×400, arrows indicate vessels) and quantitation of the CD31-positive percentage **(I,J)**, respectively (*n* = 3 independent experiments. Error bars: mean ± SD. *p < 0.5, **p < 0.1, ***p < 0.001). **(E)**. Representative confocal images of mouse-specific CD31^+^/human-specific CD31^+^ double-stained muscles derived from ischemic tissue injected with HA-MSCs-ECFCs (magnification ×400, yellow arrow indicates human-derived endothelial cells). **(F)**. Time course of the skin temperature in the ischemic limbs of the mice. **(G)**. Ambulatory impairment was scored at each time point: plantar/toe flexion to resist gentle tail traction; 1: plantar but not toe flexion; 2: no plantar or toe flexion; and 3: no use of foot. **(H)**. Blood perfusion is presented as the ratio of blood flow in the ischemic limb divided by that in the normal hindlimb. (*n* = 3 independent experiments. Scale bar represents 50 μm. Error bars: mean ± SD. *p < 0.5, **p < 0.1, ***p < 0.001 vs. the control group, ∮*p* < 0.5, ∮∮*p* < 0.01, ∮∮∮*p* < 0.001 vs. the HA group, #*p* < 0.5 vs. the MSC-ECFC group).

Next, we established mouse hindlimb ischemia models to verify that HA could improve the neovascularization of MSCs-ECFCs *in vivo*. Ischemia was induced in the right hindlimb (Figure 3A). Subsequent perfusion speckle imaging (PSI) verified that the blood flow in the ischemic right hindlimb was lower than that in the control left hindlimb after surgery (Figure 3B). PBS, HA, MSCs-ECFCs or HA-MSCs-ECFCs were injected into the ischemic sites intramuscularly 1 day after surgery. PSI showed that HA injection did not enhance blood flow compared with PBS injection throughout the observation period. Although both MSC-ECFC and HA-MSC-ECFC treatment enhanced blood flow recovery in response to HLI, the HA-MSC-ECFC group displayed a quicker recovery rate than the MSC-ECFC group 7 days after surgery (Figures 3B,H). Consistently, the mice treated with HA-MSCs-ECFCs showed rapid recovery of the ambulatory impairment index and skin temperature (Figures 3F,G).

Neovascularization in ischemic muscles was evaluated by immunofluorescence staining of CD31-positive capillaries. Increased numbers of host vessels in the ischemic muscles of the MSC-ECFC group were observed compared with those in the vehicle group. As expected, the density of host vessels was further increased by HA coadministration (Figures 3C,I). Besides, we noticed that MSC-ECFC injection did not significantly improved human-derived vessels density compared with control groups while HA-MSC-ECFC coadministration did (Figures 3D,J), which indicated that HA improved human-derived cells retention. Mouse-specific CD31^+^/human-specific CD31^+^ double staining verified that human-derived cells grew into the host vasculature and formed connections in muscles injected with HA-MSCs-ECFCs (Figure 3E). The increased numbers of CD31^+^ vessels provide a histological basis for the increased blood flow and tissue viability in the ischemic limb. These results clearly indicated that HA-MSCs-ECFCs contributed to highly effective functional blood vessel formation, leading to improved therapeutic angiogenesis for the treatment of critical limb ischemia.

### HA-MSC-ECFC Administration was Associated With CD44/miR-139-5p Signaling in Ischemic Tissue

**FIGURE 4:**
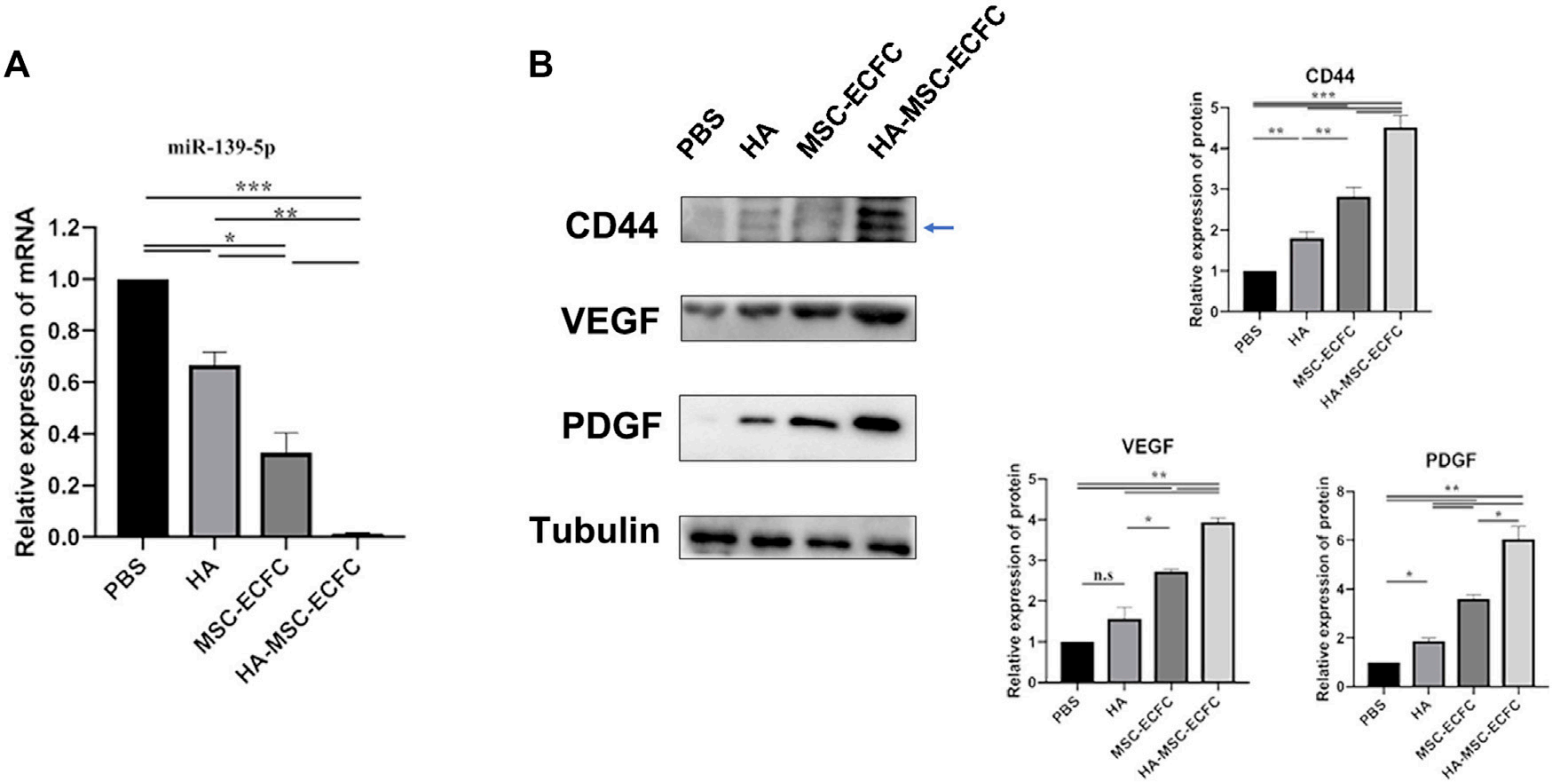
CD44 and miR-139-5p expression in ischemic tissue. **(A)**. Relative miR-139-5p expression and **(B)** relative CD44, VEGF and PDGF protein expression in the gastrocnemius muscle of the ischemic lower extremity (*n* = 3 independent experiments. Error bars: mean ± SD. *p < 0.5, *p < 0.01, *p < 0.001).

Previous studies revealed that CD44 could mediate HA-induced inhibition of miR-139-5p in ovarian cancer cells (Zhao et al., 2014). Recently, we reported that miR-139-5p mediates the impaired function of diabetic ECFCs(Luo et al., 2020). Thus, HA may enhance angiogenesis of MSCs-ECFCs through the CD44/miR-139-5p pathway. Next, we explore the mechanism of HA enhancing angiogenesis of MSCs-ECFCs in the ischemic limb. We assessed the expression levels of CD44, miR-139-5p and the downstream angiogenesis-related molecules VEGF and PDGF in ischemic muscles. MSC-ECFC administration significantly decreased the level of miR-139-5p (Figure 4A) while improving the levels of CD44, VEGF and PDGF (Figure 4B) in ischemic muscles compared with control, and HA coadministration further enhanced this effect. These results indicated that HA enhances angiogenesis of MSCs-ECFCs, probably by activating CD44 and repressing the miR-139-5p pathway.

### Downregulation of miR-139-5p Expression was Induced by HA by Activating CD44 and was Involved in HA Promotion of Angiogenesis

**FIGURE 5:**
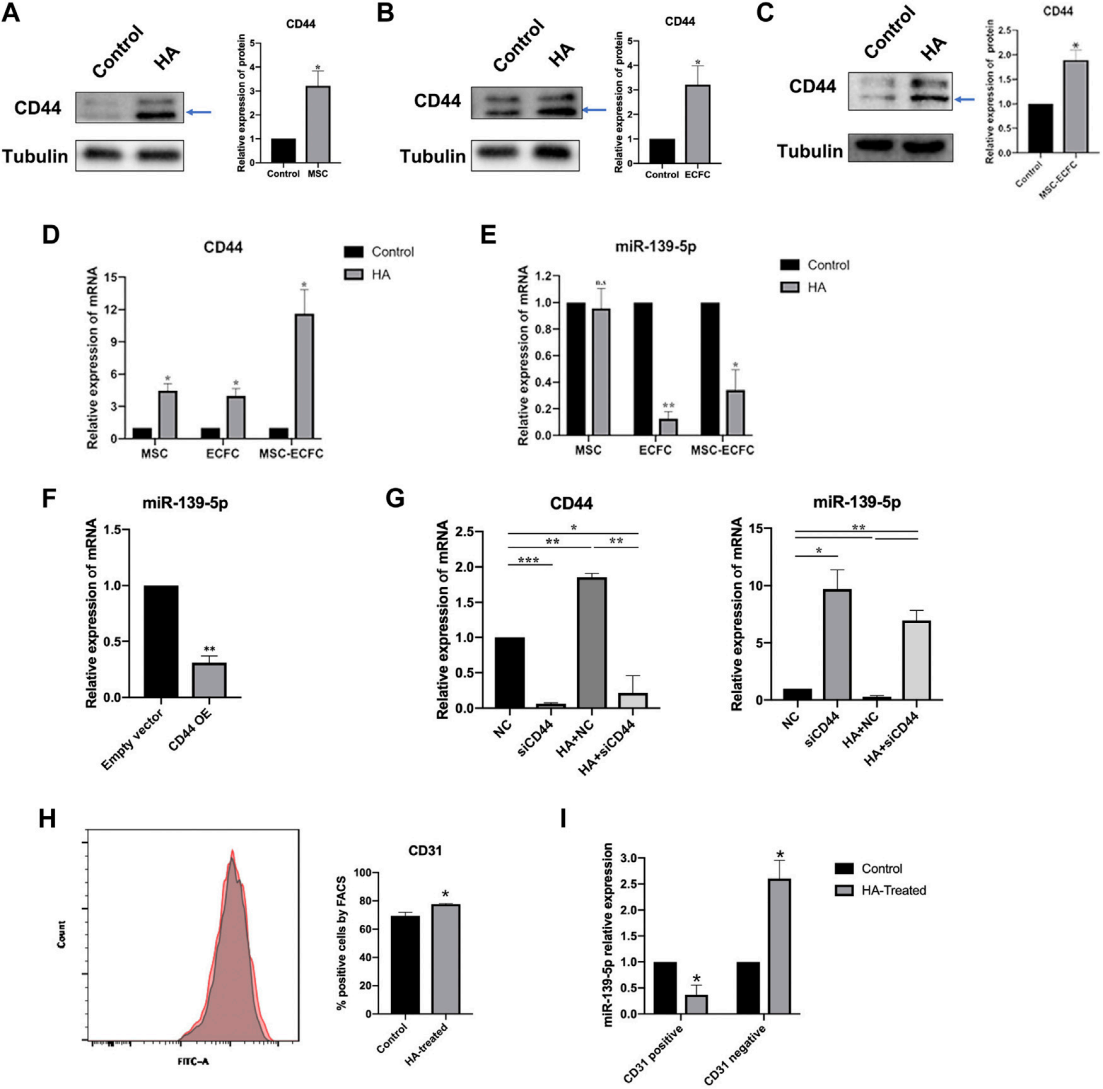
HA inhibits miR-139-5p expression by activating CD44. **(A–C)**. Western blot assays and qPCR assays **(D,E)** were performed to test CD44 and miR-139-5p expression after HA treatment in MSCs, ECFCs and MSCs-ECFCs, respectively. **(F)**. Relative miR-139-5p expression in ECFCs transfected with CD44 overexpression plasmid. **(G)**. qPCR assays were performed to test the CD44 and miR-139-5p mRNA expression in ECFCs transfected with CD44 siRNA, with or without administration of HA, respectively. **(H)**. Flow cytometry of CD31 sorted from MSC-ECFC co-cultured system, with (red) or without (grey) administration of HA, and quantification of flow cytometry. **(I)**. Relative miR-139-5p expression in the sorted CD31^+^ cells and CD31^−^cells, respectively. (*n* = 3 independent experiments. Error bars: mean ± SD. *p < 0.05, **p < 0.01, ***p < 0.001).

To clarify the effect of HA on the expression of miR-139-5p in MSCs and ECFCs, we tested the expression of CD44 and miR-139-5p with or without HA dilution in MSCs, ECFCs and cocultured MSCs-ECFCs, respectively. As shown in Figure 5, the expression of CD44 was increased in different type of cells after exposure to HA (Figures 5A–D). The level of miR-139-5p was significantly decreased in the ECFCs and MSCs-ECFCs treated with HA, while no change of miR-139-5p expression in MSCs alone was observed (Figure 5E). Then, we transfected CD44 plasmids to overexpress CD44 (Supplementary Figures S1A,B) in ECFCs and found that miR-139-5p expression was significantly repressed (Figure 5F). Knockdown of CD44 expression through transfection with small interfering RNAs (siRNAs) in ECFCs (Supplementary Figure S1C) effectively enhanced miR-139-5p expression and abrogated the HA-mediated downregulation of miR-139-5p expression (Figure 5G). These results indicated that CD44 is a negative regulator of miR-139-5p and that HA could decrease miR-139-5p expression through CD44 in ECFCs. Since HA did not regulate miR-139-5p expression in MSCs, we wondered whether it would make affect in the co-culture system. We sorted CD31^+^ and CD31^−^cells in MSC-ECFC co-culture mixed cells using flow cytometry, respectively. Results showed that the proportion of CD31^+^ cells was significantly higher in the HA-treated group (Figure 5H). Moreover, the significantly decreased miR-139-5p expression in the CD31^+^ group (Figure 5I) explained the reduced overall expression of miR-139-5p in the HA-MSC-ECFC group.

**FIGURE 6:**
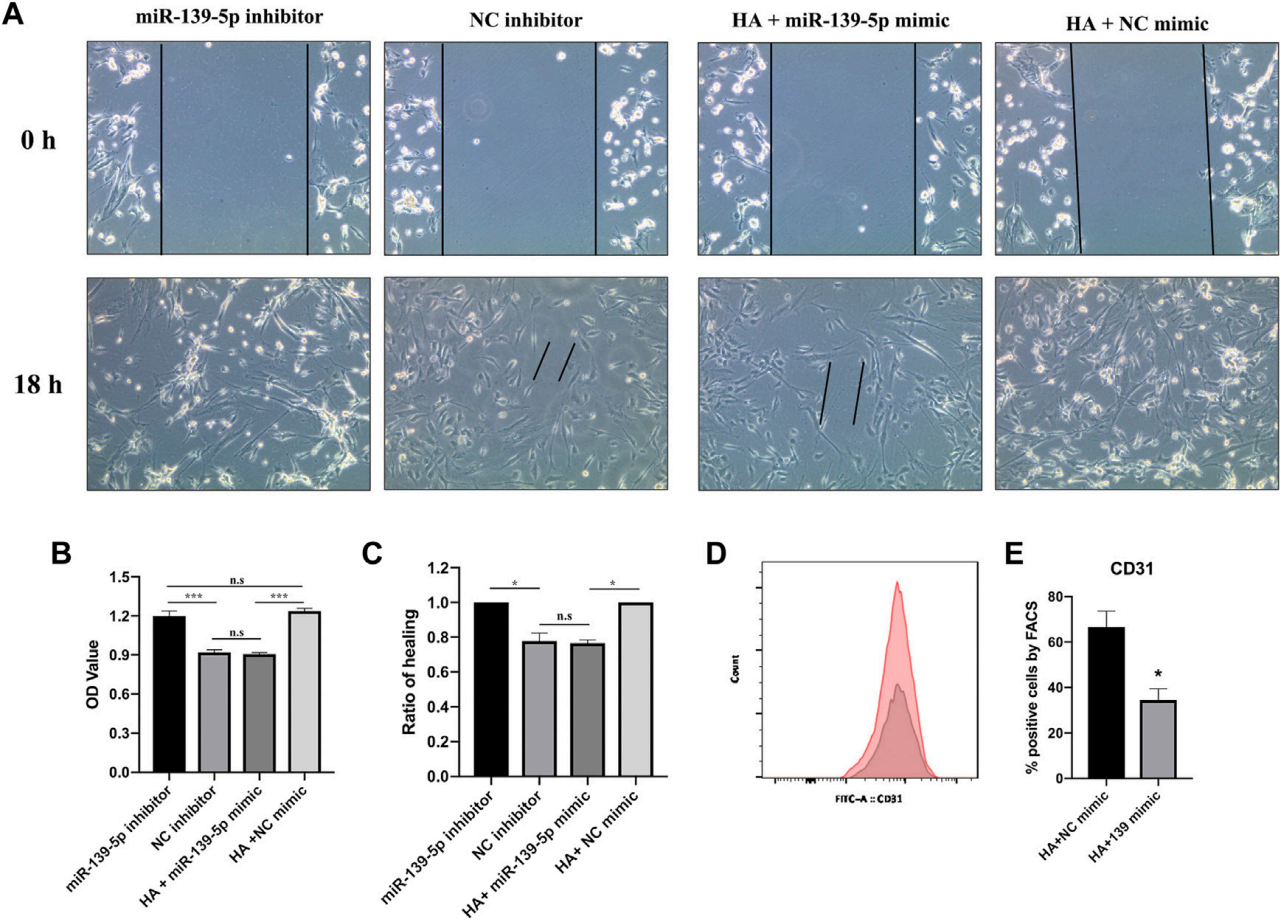
HA promotes MSCs-ECFCs proliferation and migration through miR-139-5p. **(A,C)**. Wound healing assays were performed to assess the migration of MSCs-ECFCs transfected with miR-139-5p mimics and HA-MSCs-ECFCs transfected with miR-139-5p inhibitors, respectively. **(B)**. CCK8 assays were performed to assess the proliferation. **(D)**. Flow cytometry of CD31 sorted from HA-MSC-ECFC transfected with NC mimics (red) or miR-139-5p mimics (grey). **(E)**. Quantification of flow cytometry. (*n* = 3 independent experiments, Error bars: mean ± SD. *p < 0.05, **p < 0.01, ***p < 0.001).

To determine whether miR-139-5p is involved in HA-mediated promotion of MSCs-ECFCs function, we transfected MSCs-ECFCs with miR-139-5p inhibitors and transfected HA-MSCs-ECFCs with miR-139-5p mimics, respectively (Supplementary Figures S1D,E). Results showed that upregulation of miR-139-5p expression dampened the HA-improved MSC-ECFC proliferation and migration; in contrast, downregulation of miR-139-5p expression significantly improved MSC-ECFC proliferation and migration (Figures 6A–C). Furthermore, we found that the proportion of CD31^+^ cells was significantly reduced in HA-MSC-ECFC transfected with miR-139-5p mimics (Figures 6D,E), which indicated that HA regulate ECFCs viability in the HA-MSC-ECFC co-culture system through miR-139-5p. In conclusion, these results suggested that HA promotes angiogenesis of MSCs-ECFCs at least partly through the CD44/miR-139-5p pathway.

## Discussion

Therapeutic angiogenesis is an emerging approach to reconstruct the damaged vascular network by promoting local angiogenesis and stimulating *de novo* blood vessel formation. Among these approaches, cell therapy is a promising strategy. ECFCs are a subset of EPCs that arise from circulation. In particular, human cord blood-derived ECFCs have a high proliferative capacity and potently form stable and functioning blood vessels *in vivo* (Jokubaitis et al., 2008). We and others have proposed that ECFC significantly rescues blood perfusion at 14 days post-injection by vascular incorporation (Schwarz et al., 2012; Luo et al., 2020). Some studies have revealed that combining MSCs at a certain ratio with ECFCs enables the formation of lasting microvascular networks that anastomose with the host vasculature following transplantation *in vivo* (Melero-Martin et al., 2008) and improves blood flow in ischemic limbs compared with ECFC or MSC administration alone (Creager et al., 2012; Kang et al., 2017). Generally, the interaction between endothelial cells and mural cells (pericytes and vascular smooth muscle) is essential for vascular development and maintenance. ECFCs can differentiate into endothelial cells in ischemic tissues (Critser and Yoder, 2010; O'Neill et al., 2018), while MSCs can differentiate into perivascular cells, which envelop endothelial cells in the vascular structure (Melero-Martin et al., 2008). In addition, MSCs secrete a plethora of proangiogenic factors, such as VEGF-A and FGF2, to induce the proliferation, migration, and tube formation of ECFCs, thereby rescuing local blood perfusion (Lin et al., 2012). Other mechanisms of MSC-synergized ECFCs in angiogenesis include modulated ECFC angiogenic activity *via* direct contact by priming ECFCs toward a mesenchymal phenotype, which enhances adhesive and tubulogenic properties (Shafiee et al., 2017), potentiates ECFC retention by recruiting host myeloid cells (Kang et al., 2017) and reduces intragraft immune cell infiltration and endothelial HLA-DR expression, thus lowering the risk of ECFC rejection (Souidi et al., 2017). A previous study suggested that mixing MSCs-EPCs at a ratio of 60:40 increased vessel density. The present study demonstrated that MSC-ECFC (3:2) cotransplantation formed more vascular networks in Matrigel plugs than MSC or ECFC transplantation alone.

Concomitant administration of MSCs and ECFCs was safe and had synergistic effects on angiogenesis and tissue regeneration (Shah and Kang, 2018), but the unsatisfactory treatment efficiency due to low engraftment and survival after transplantation was a concern (Karp and Leng Teo, 2009; Moubarik et al., 2011). To improve this situation, researchers have assessed new compounds as injectable and biological vehicles for delivery (Heo et al., 2014; Goto et al., 2016; Ullah et al., 2019). The injection of biomaterials combined with stem cells represents a promising solution to treat ischemic disorders (Prestwich et al., 2012). HA is an extracellular matrix component with good biocompatibility, biodegradability, and nonimmunogenicity and exerts antioxidative, anti-inflammatory, and analgesic effects *in vivo*. As a physiological component, it is not expected to produce adverse reactions after administration in clinical trials (Bowman et al., 2018). Therefore, HA is currently applied in arthritis therapy, medical cosmetology and tissue engineering (Gupta et al., 2019; López-Ruiz et al., 2019). Zullo et al. recently showed that HA-based hydrogen-coembedded EPCs and MSCs alleviated inflammation and improved cell viability *in vivo* (Zullo et al., 2015). Our previous work revealed that HA, MSCs and ECFCs could safely synergize to accelerate the healing of refractory diabetic foot ulcers (Zhao et al., 2020). These results indicated that HA-MSC-ECFC treatment may be a promising strategy for clinical use. Here, we used Matrigel plug assays to test *in vivo* angiogenesis. Clearly, HA enhanced angiogenesis of MSCs, ECFCs and MSCs-ECFCs, as more capillaries and vasculature with large diameters were observed in the HA-MSC-ECFC plugs. Moreover, HA-MSC-ECFC administration in mouse ischemic muscle accelerated blood perfusion and promoted neovascularization in tissues. These results suggested that HA-MSC-ECFC combined therapy may be a promising strategy for ischemic diseases.

Consistent with other research (Liu et al., 2016; Corradetti et al., 2017; Wang et al., 2020), the present work demonstrated that HA promotes MSC proliferation and migration. To the best of our knowledge, no data concerning the influence of HA on ECFCs have been reported. Our study showed that HA promoted the migration of ECFCs without damaging proliferation. Moreover, we found that HA significantly improved the migration and proliferation of cocultured MSCs-ECFCs. To choose the appropriate concentration, we tested 0.5 mg/ml and 1 mg/ml concentration of HA dilution. Both concentration of HA induced a positive effect on cell migration and proliferation. Co-cultured MSCs-ECFCs displayed no significance in proliferation and between two HA concentration groups. Therefore, we used the concentration of 0.5 mg/ml HA in the subsequent study. Mechanistically, HA regulates cell proliferation and migration by targeting specific receptors such as CD44 (Tavianatou et al., 2019; Skandalis et al., 2019). HA was shown to improve MSC survival, proliferation and migration by targeting CD44 (Corradetti et al., 2017). Previous work proved that ECFCs with high CD44 expression were more effective at facilitating regeneration of retinal vasculature than those with lower CD44 expression in an oxygen-induced retinopathy model (Sakimoto et al., 2017). The present study showed that HA promoted CD44 expression in MSCs, ECFCs and cocultured MSCs-ECFCs, respectively, which indicated that HA may improve MSC-ECFC function by targeting CD44.

Interestingly, HA-CD44 interactions regulate tumor progression by activating miRNAs(Bourguignon, 2019). Several studies have demonstrated that CD44 could inhibit miR-139-5p expression in tumor cells (Bao et al., 2011; Zhao et al., 2014). miR-139-5p regulated the biological process of cells by targeting a variety of target genes, such as c-Jun (AP-1), IGF-1R, Notch, CXCR4, etc. Growth factors such as VEGF, PDGF and c-jun are regulated by miR-139-5p in endothelial cells (Zhang et al., 2021). Here, we found that the levels of CD44, VEGF and PDGF were increased, while miR-139-5p expression was decreased in ischemic muscles treated with HA-MSCs-ECFCs compared with that in the MSC-ECFC group. *In vitro* assays revealed that miR-139-5p expression was downregulated in ECFCs and cocultured MSCs-ECFCs treated with HA dilution. miR-139-5p expression negatively regulated by CD44 in ECFCs showed by the gain- or loss-function experiments. Precious study revealed that conditional medium derived from MSC increased CD31 expression in EPC(Fauza et al., 2018). In the present study, flow cytometry assays showed that CD31 positive cells rose to 60% in MSC-ECFC group, verified that coculture with MSCs improved ECFC viability. Although HA did not improve ECFCs proliferation when monoculture, CD31 positive cells further increase (77.6%) in HA-MSC-ECFC coculture group demonstrated that HA significantly enhanced ECFCs viability in MSC-ECFC co-culture system. These results suggest that all three ingredients were important and indispensable in the HA-MSC-ECFC system. Besides, we found that enhanced proportion CD31^+^ cell in HA-MSC-ECFC co-cultured system could be reversed by upregulation of miR-139-5p. We considered that HA improved ECFCs viability through miR-139-5p in the HA-MSC-ECFC co-culture system. Generally, MSC could directional differentiated into endothelial cells in the medium supplemented with cytokines (Wang et al., 2018; Zhang et al., 2020) and miRs participate in the process (Arderiu et al., 2019). Decreased miR-139-5p expression in CD31 positive cells induced by HA may regulate target genes expression such as increased expression of VEGF and PDGF, which may further induced endothelial differentiation and proliferation. However, whether the increased CD31 positive proportion may partly due to the MSC endothelial differentiation or downregulation of miR-139-5p reflects differentiation of MSCs leaves us an intriguing question and need further research. On the other hand, we noticed that miR-139-5p expression was increased in CD31 negative cells in the HA-MSC-ECFC coculture system, which could inhibits MSCs proliferation (our unpublish data) and also may be associated with decreased MSC proportion in coculture system. Moreover, we found that upregulation of miR-139-5p expression could dampen HA-improved MSCs-ECFCs proliferation and migration, while downregulation of miR-139-5p expression improved MSCs-ECFCs proliferation and migration. These results indicated that HA promoted MSC-ECFC angiogenesis through the CD44/miR-139-5p pathway.

In summary, the present study demonstrates that HA facilitates angiogenesis of MSCs-ECFCs, revealing new mechanistic insights into the CD44/miR-139-5p signaling pathways involved, and provides novel and practical treatment approaches for vascular diseases.

## Data Availability

The raw data supporting the conclusion of this article will be made available by the authors, without undue reservation.
