# Supplementary figures and images for: Hyaluronic Acid Facilitates Angiogenesis of Endothelial Colony Forming Cell Combining With Mesenchymal Stem Cell via CD44/ MicroRNA-139-5p Pathway
Source: Front Bioeng Biotechnol. 2022 Mar 8;10:794037. doi: 10.3389/fbioe.2022.794037 (PMC8957954; doi:10.3389/fbioe.2022.794037)

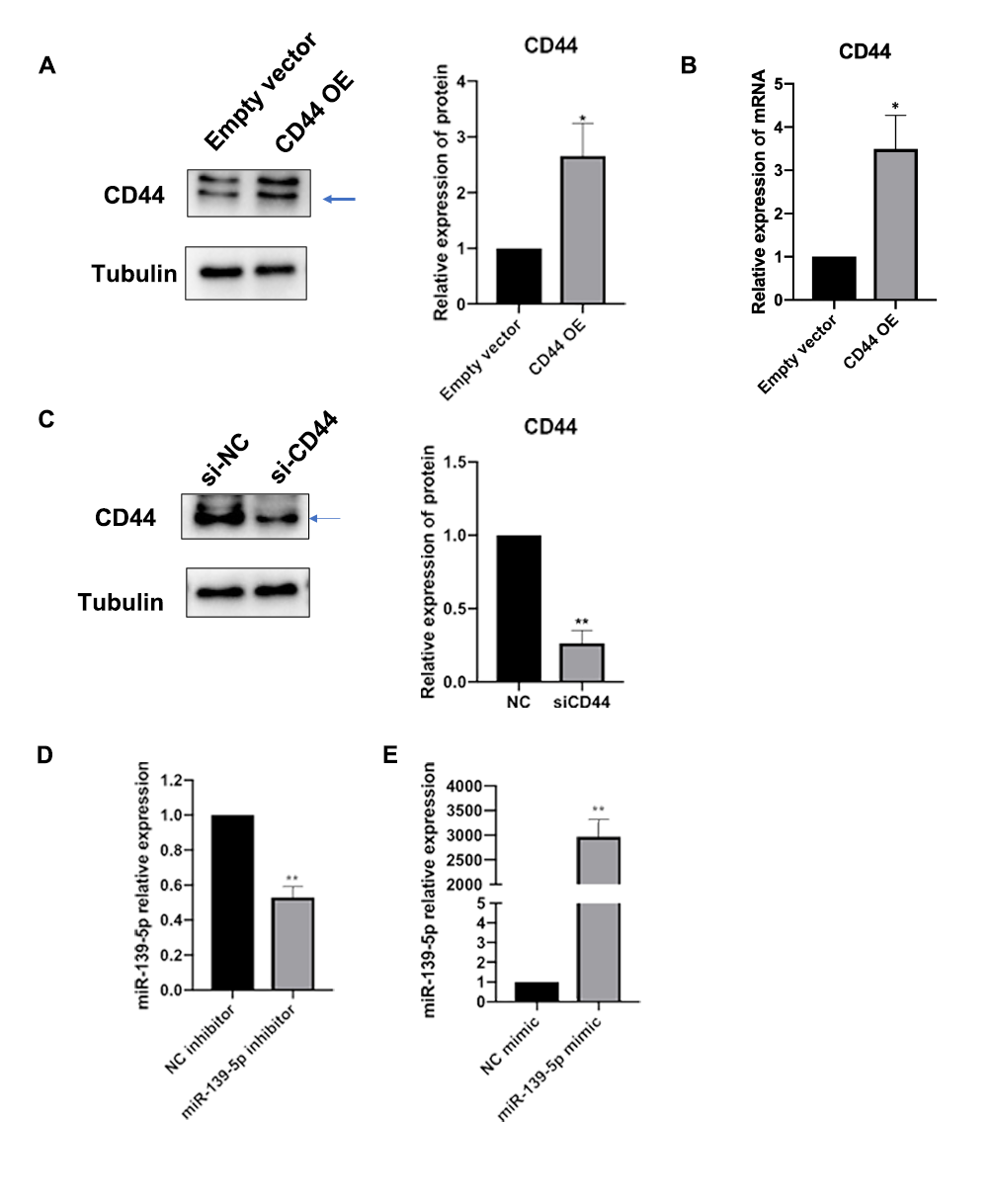

Supplement: Supplementary file 1 [file Image1.tif]
